# Template-Directed RIG-I Agonist Assembly for Image-guided Targeted Cancer Immunotherapy

**DOI:** 10.1007/s11307-026-02087-8

**Published:** 2026-02-19

**Authors:** Subrata K. Ghosh, Douglas Lazarus, Neil Robertson, Qiyong P. Liu, Elizabeth Kenyon, Christian L. Mallett, Ming Chen, Zdravka Medarova, Anna Moore

**Affiliations:** 1 TransCode Therapeutics, Inc., 400 Trade Center, Suite 5900, Woburn, MA 01801 USA; 2 Avastus Preclinical Services, 44 Spinelli Place, Cambridge, MA 02138 USA; 3 Precision Health Program, Michigan State University, 766 Service Rd., East Lansing, MI 48224 USA; 4 Department of Radiology, College of Human Medicine, Michigan State University, 846 Service Rd., East Lansing, MI 48224 USA; 5 Institute for Quantitative Health Science and Engineering, Michigan State University, 775 Woodlot Dr., East Lansing, MI 48224 USA

**Keywords:** Immunotherapy, Image-guided therapy, RIG-I signaling, Oligonucleotide delivery

## Abstract

**Purpose:**

Tumor-specific immunotherapies selectively target tumor cells with reduced toxicity compared to conventional treatments. Pattern recognition receptors, such as retinoic acid-inducible gene I (RIG-I)-like receptors, have been used to induce broad antitumor responses but their off-target effects and delivery issues hinder their clinical translation. To overcome these challenges, we present a strategy that involves the intracellular assembly of the RIG-I agonist on a tumor-specific RNA template (e.g., miRNA-21) by delivering a 5'-triphosphate single-stranded RNA RIG-I agonist (RIGA-miRNA-21) by a superparamagnetic nanoparticle carrier (TTX) to initiate specific RIG-I signaling and antitumor immune responses. Magnetic properties of TTX enable its detection by magnetic resonance imaging (MRI) supporting the concept of image-guided therapy.

**Procedures:**

A single-stranded anti-miR-21 5’-triphosphate RIG-I agonist was conjugated to the dextran coat of the nanoparticles through disulfide bonds producing TTX-RIGA-miR-21 and tested *in vitro* and *in vivo* in B16-F10 melanoma model. Delivery of the TTX carrier was demonstrated in mice bearing B16-F10 tumors by MRI. Therapeutic studies included intravenous injections of TTX-RIGA-miR-21 or controls for 7 days starting on Day 4 after tumor implantation. On Day 15, animals were rechallenged with additional B16-F10 cells implanted on the opposite side.

**Results:**

We demonstrated that TTX-RIGA-miR21 was able to induce miRNA-21-dependent RIG-I signaling and apoptosis in melanoma cells, inhibit tumor growth, and induce immunity against tumor rechallenge in an animal model.

**Conclusions:**

Our template-driven approach brings RIG-I closer to becoming a clinically relevant target in oncology by specifically activating immune responses within tumor cells through systemic RIG-I agonist delivery.

**Supplementary Information:**

The online version contains supplementary material available at 10.1007/s11307-026-02087-8.

## Introduction

Tumor-specific immunotherapy holds significant clinical promise owing to its potential to precisely eliminate malignant tumors without adding toxicity to standard treatments. Immune checkpoint inhibitors (ICIs) have demonstrated remarkable success in the treatment of diverse tumor types. However, they often fail in ‘immune-cold’ tumors with low levels of tumor-infiltrating lymphocytes [1].

Pattern recognition receptors (PRRs) recognize specific evolutionarily conserved pathogen structures called pathogen-associated molecular patterns. When PRRs engage with these structures, they activate intracellular signaling cascades, resulting in the release type I interferons (IFNs), which induces interferon-stimulated genes (ISGs) and proinflammatory cytokines. This orchestrates the initial host response to infection and sets the stage for subsequent development of adaptive immunity [2].

Inspired by innate immunity against microbes, cancer immunotherapy targeting PRRs, such as retinoic acid-inducible gene 1-like receptors (RIG-I-like receptors, RLRs), has emerged [3]. RLRs are vital cytosolic RNA sensors in human cells that detect viral infections and initiate antiviral immune responses. Introducing a short 5'-triphosphate double-stranded RNA (5’-ppp-dsRNA) can simulate viral infection and direct the immune responses towards cancer cells. Activation of RLRs in tumors has been found to cause tumor cell death and activation of innate immunity in the tumor microenvironment (TME) [4, 5]. Synthetic PRR agonists are currently under investigation in preclinical and early clinical trials for gliomas, multiple myeloma, breast, pancreatic, and ovarian cancers [4–10]. However, the majority of these studies employ double-stranded constructs such as siRNA or stem-loop RNAs that adopt a double stranded secondary structure upon folding. Delivery of such constructs to tumor cells either in their unformulated form or using carriers is non-specific. Since RIG-I-like receptors are ubiquitously expressed in the body [6, 7], these agonists can elicit a non-specific response, leading to significant toxicity and undesired systemic immune activation. In addition, most studies investigating RIG-I agonists have been conducted in models of intratumoral delivery [8, 11] which in most cases lacks clinical significance. The use of other delivery methods such as the cationic polymeric transfection reagent jetPEI® in clinical [10] and preclinical studies [6–9], as well as PLGA polymers [12], lipid calcium phosphate [13], lipid-based nanoparticles [14], and extracellular vesicles [15] still did not offset the issue of non-specific activation of RIG-I by double-stranded constructs.

In this work we addressed both issues by introducing a model of selective activation of RIG-I in cancer cells, directed by an overexpressed oncogenic miRNA. Unlike conventional methods using RIG-I agonists, 5’-triphosphate double stranded RNA (5’-ppp-dsRNA) or 5’-ppp-RNA hairpins [8–10, 16], our approach employs a single-stranded “inactive” RIG-I agonist, 5’-triphosphate antisense miRNA oligonucleotide with perfect complementarity to a prevalent miRNA in tumor cells. Systemic delivery of this RIG-I agonist was achieved using dextran-coated iron oxide nanoparticles (TTX platform), which served as vehicles for oligonucleotide delivery to tumor cells *in vivo*. A single-stranded 5’-triphosphate RIG-I agonist is attached to the dextran coat of the nanoparticles through disulfide bonds [17]. The magnetic properties of these nanoparticles enabled their detection by magnetic resonance imaging (MRI), allowing non-invasive monitoring of payload delivery and supporting the concept of image-guided therapy. We have previously used these nanoparticles as a platform for oligonucleotide delivery and imaging in breast cancer [17–19], pancreatic cancer [20] and glioblastoma [21, 22] in small and large animals [17–19, 23].

**Fig. 1:**
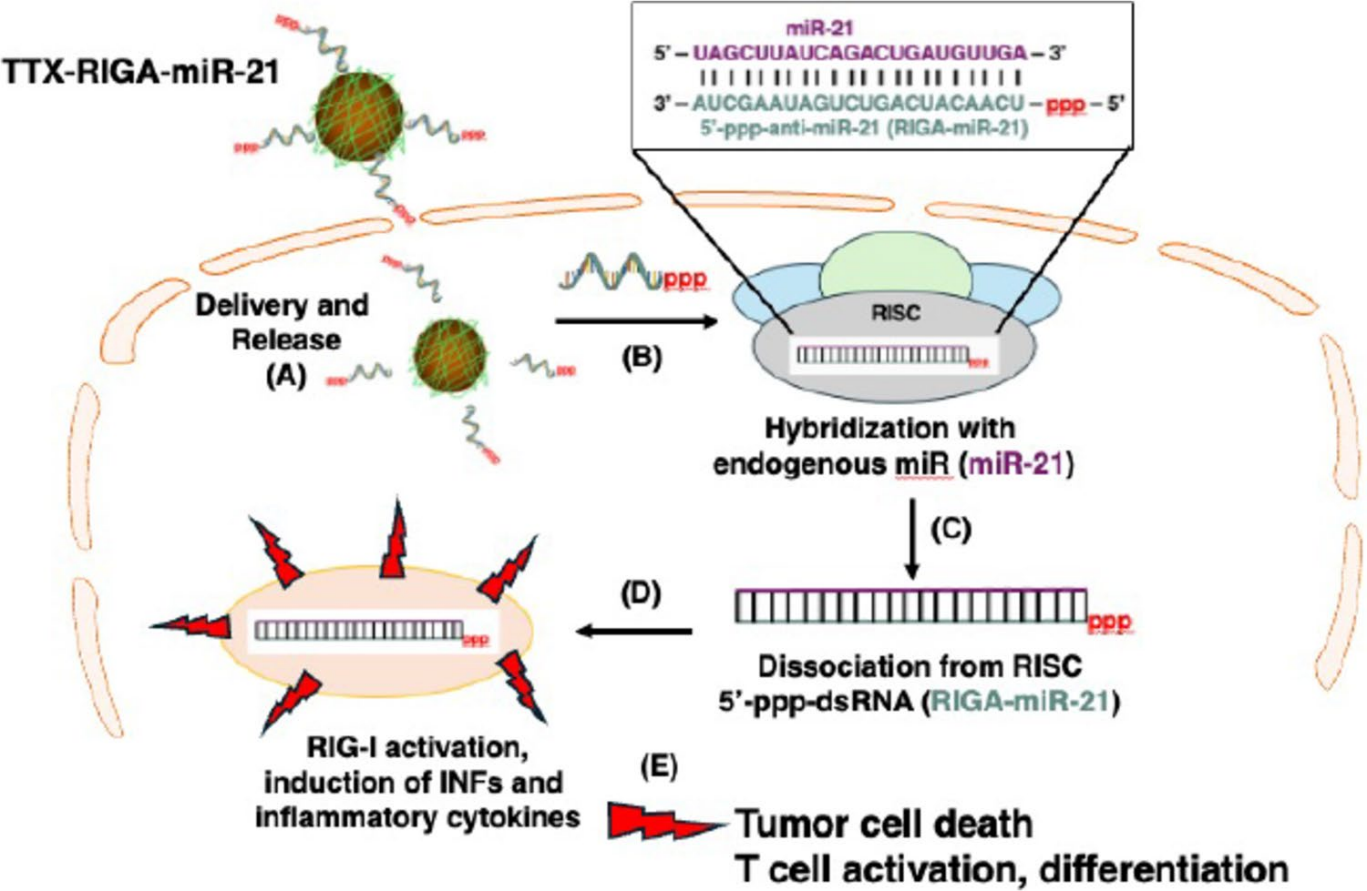
Design and a working model of the template-specific RIG-I agonist TTX-RIGA-miR-21. **A**. Upon entering the cell and cleavage of disulfide bonds, RIGA-miR-21 dissociates from the TTX nanocarrier. **B**. Dissociated RIGA-miR-21 binds to endogenous single stranded miRNA-21, forming standard RIG-I agonist 5'-ppp-dsRNA. **C**. Dissociation from the RNA-induced silencing complex (RISC). Alternatively, RIGA-miR-21 can also form complexes with free single stranded miRNA-21 outside of the RISC. **D**. RIG-I activation following detection of the complex in the cytosol; release of IFNs and pro-inflammatory cytokines. **E**. Anti-tumor response, tumor cell death

The proposed design and mechanism of action of the template-specific RIG-I agonist is shown in Fig. 1. As the template for our RIG-I agonist, TTX-RIGA-miR-21, we selected miRNA-21, commonly abundant in tumor cells [24, 25]. However, since RIG-I agonism is independent of the dsRNA sequence [26] this concept could be applied to other miRNA targets enriched in tumor cells. TTX-RIGA-miR-21 was designed to be 100% complementary to miRNA-21, with a 5'-ppp modification (Fig. 1, inset). Upon entering the cell and cleavage of disulfide bonds (Fig. 1A), RIGA-miR-21 dissociates from the TTX nanocarrier and binds to endogenous single stranded miRNA-21, forming standard RIG-I agonist 5'-ppp-dsRNA (Fig. 1B). Next, this complex likely dissociates from the RNA-induced silencing complex (RISC), as observed before [27] (Fig. 1C). Alternatively, RIGA-miR-21 can also form complexes with free single stranded miRNA-21 outside of the RISC [28]. RIG-I then detects this complex in the cytosol, leading to its activation (Fig. 1D). This activation, known for its immune-modulating effects [29] triggers the production of type I IFNs and inflammatory cytokines, causing cancer cell death (Fig. 1E). Subsequently, the release of IFNs, pro-inflammatory cytokines and tumor antigens remodels the tumor microenvironment (TME) [30]. Naïve T cells are activated, proliferate, differentiate, and migrate to the TME, exerting the antitumor responses through cytolytic activity and cytokine secretion like IFN-γ and TNF-α. After cancer cell death, most tumor specific CD8^+^ T cells perish, but some become long-lasting memory CD8^+^ T cells (Fig. 1E).

In the studies described here we validated this approach by testing TTX-RIGA-miR21 *in vitro* and in a mouse melanoma model. We first showed that the formulation of a double stranded 5’-ppp complex triggered RIG-I signaling *in vitro* and induced the expression of cancer cell-derived cytokines (IFN-β and IP-10/CXCL10), representative biomarkers of immunogenic cell death caused by RIG-like helicase ligands [29, 31], as well as induction of apoptosis. Further, we confirmed the delivery of the nanocarrier to the tumors by *in vivo* imaging after systemic injection and performed therapeutic studies in a mouse melanoma model. We showed that TTX-RIGA-miR21 elicited a noteworthy 'abscopal effect' on secondary tumors, and induced IFN-β expression in both primary and secondary tumors *in vivo*. In this work we focused on melanoma as one of the deadliest cancers with poor survival [32]. Considering that the nanoparticles used in this study represent a multifunctional platform which can be used for conjugation with multiple oligonucleotide moieties, these results can be extended to other cancers with overexpressed miRNAs. Furthermore, given that TTX-based nanoformulations developed by our group are already in clinical trials (NCT06260774), the clinical translation of these findings is highly feasible.

## Materials and Methods

### Oligonucleotides

The miR-21 mimic, 5’-UAGCUUAUCAGACUGAUGUUGA-3', and its 100% complementary antisense complement, [± (ppp)]−5’-UCAACAUCAGUCUGAUAAGCUA-3’-(s–s) (termed RIGA-miR-21), were synthesized by Eurogentec (Fremont, CA). 5’-Triphosphate modification (ppp) is required for RIG-I agonism while 3’ disulfide bond (s–s) modification allows the oligos to be covalently conjugated to the nanoparticles. Antisense oligonucleotide (ASO) for miR-21 (anti-miR-21) was synthesized as well. A synthetic RIG-I agonist, 19-mer 5'-ppp-dsRNA, was obtained from InvivoGen (Catalog No. tlrl-3prna; San Diego, CA).

### Cell Culture and Transfection

HEK-Lucia™ RIG-I cells, HEK-Lucia™ Null cells (Catalog No. hkl-hrigi and hkl-null; InvivoGen), or murine melanoma cells (B16-F10) were cultured in 96-well plates for 48 h in Dulbecco's Modified Eagles Medium (DMEM, Gibco; ThermoFisher Scientific, Waltham, MA) containing 10% fetal bovine serum, 100 U/mL of penicillin and 100 µg/mL of streptomycin at 37 °C, 5% CO_2_. Murine 4T1 murine triple negative breast cancer cells were cultured in RPMI-1640 (ThermoFisher Scientific, Waltham, MA) supplemented with 10% fetal bovine serum, 100 U/mL of penicillin and 100 µg/mL of streptomycin at 37 °C, 5% CO_2_. Cells were transfected using LyoVec™ cationic lipid transfection agent (see Supplementary Materials for details). In all studies miR‑21 mimic concentration was held constant across all nanoparticle doses to ensure changes in signaling reflected nanoparticle-dependent oligo delivery rather than variable template abundance.

### Nanoparticle Formulation of RNA Oligos

The synthesis of oligo-nanoparticle conjugates, TTX-RIGA-miR-21 and TTX-miR-21 and their characterization followed a previously described protocol with some methodological modifications [18] (see Supplementary Materials for details).

For *in vivo* optical imaging and ex-vivo fluorescence microscopy nanoparticles were labeled with the Cy5.5 near infrared fluorescence dye as described in our earlier publications [17, 18].

### Western Blotting

To assess protein expression of IFN-β, IP-10, phospho 65/65 and RIG-I we performed Western blot with corresponding antibodies (see Supplementary Materials for details).

### RIG-I Activation Assay

Twenty µL of culture media from transfected cells was transferred to a 96-well clear-bottom black plate, 50 µL of QUANTI-Luc™ assay solution (Catalog No. rep-qlc, InvivoGen) was added to each well. Luminescence was immediately measured using a Spectramax M3 microplate reader (Molecular Devices, San Jose, CA) set at a 0.1 s of exposure. Data are presented as mean ± SD.

### IFN-γ-inducible Protein 10 (IP-10/CXCL-10) Immunoassay

IP-10/CXCL10 release was assayed using the Quantikine Mouse IP-10 ELISA assay (DY466-05; R&D Systems, Minneapolis, MN) using cell-free media following the manufacturer’s instructions. The absorbance at 450 nm was measured, and the concentration of IP-10 was determined by comparison to the standards. All experiments were performed in triplicate. Data are presented as mean ± SD.

### MTT Assay

The Cell Proliferation Kit I (MTT) (Product No. 11465007001; Fisher Scientific, Hampton, NH) was utilized following the manufacturer’s instructions. Ten μL of MTT was added to each cell culture and incubated for 4 h in a humidified atmosphere (e.g., 37 °C, 5% CO_2_). Then, 100 μL of the solubilization solution was added to each well and the plate was kept overnight in the incubator. The absorbance of the formazan product was determined at 560 nm with a reference wavelength set at 670 nm.

### Caspase-3/7 Activation and Viability Assays

Caspase-3/7 activity was determined using the Caspase-Glo 3/7 Assay Kit (Catalog No. G8091; Promega, Madison, WI) following the manufacturer’s instructions. Cell cultures were equilibrated to RT, and 100 µL of Caspase-Glo® 3/7 Reagent was added to each well. Plates were incubated at RT for 1 h and luminescence was measured. Cell viability was determined using CellTiter-Glo® 2.0 Assay after 48 h of incubation according to manufacturer’s instructions.

### IFN-β Induction Assay

IFN-β production was analyzed using 50 µL of culture supernatant by mouse IFN-β ELISA Kit (LEGEND MAX™ Mouse IFN-β ELISA Kit, Catalog No. 439407; BioLegend, San Diego, CA) following the manufacturer’s instructions. Absorbances at 450 nm were determined.

### RT-qPCR

To assess expression of miR-21, RIG-I, IFN-β, IP-10 and TRAIL we employed RT-qPCR (see Supplementary Materials for details and list of primers).

### *In vivo *Imaging of Tumoral Delivery

Mice (C57BL/6 J, 8-week-old) were obtained from the Jackson Laboratory. B16-F10 melanoma cells (1 × 10^5^) were subcutaneously injected into the right flank. To assess the delivery, mice were imaged using *in vivo* fluorescence and magnetic resonance imaging (FLI and MRI, see Supplementary Materials for details). Quantification of nanoparticle delivery was performed by measuring tumor ROI radiance normalized to background and by calculating change in T2* relaxation time following nanoparticle administration.

### Therapeutic Studies in a Mouse Melanoma Model

Mice bearing B16-F10 tumors were randomized into three groups (*n* = 5/group). Mice were intravenously treated with TTX-RIGA-miR-21 (400 µg/kg) or PBS and intratumorally with 5’-ppp-dsRNA (1 µg/mouse). Treatments started on Day 4 after tumor implantation and continued daily for 7 d. On Day 15, additional B16-F10 cells (1 × 10^5^) were implanted on the opposite side (Supplementary Fig. S1). Tumor sizes were measured with calipers. Tumor volumes were calculated using Vtumor = (L x W2) × 0.5. Mice were euthanized when tumors reached their allowed maximum volume. Data analysis was carried out by a blinded investigator. Animal studies were approved by the Institutional Animal Care and Use Committee (IACUC) at Michigan State University and are in compliance with the National Institutes of Health Guide for the Care and Use of Laboratory Animals.

### Ex vivo Fluorescence Microscopy

After the last imaging session, animals were sacrificed, tumors were excised, snap frozen in OCT and cryosectioned into 10 µm sections. Sections were placed on glass slides, and fixed in 4% paraformaldehyde (PFA) for 15 min. The slides were then rinsed with PBS and mounted with Fluoromount-G mounting medium (Southern Biotech, Birmingham, AL, USA) containing 4',6-diamidino-2-phenylindole (DAPI). Slides were then imaged in Cy5.5 and DAPI channels using a Nikon Eclipse 50i microscope.

### Statistical Analysis

Statistical analysis was performed using GraphPad Prism 9.5.1 (GraphPad Software, San Diego, CA). Data are presented as mean ± SD or SEM. Two-tailed t-tests were used in all analyses except dose response, which was analyzed using non-linear regression. A *p* value of less than 0.05 was considered statistically significant.

## Results

### The Template-specific RIG-I Agonist Agonizes RIG-I in a Template-dependent Manner in the Reporter Cell Line and Induces Apoptosis in Melanoma Cells

**Fig. 2:**
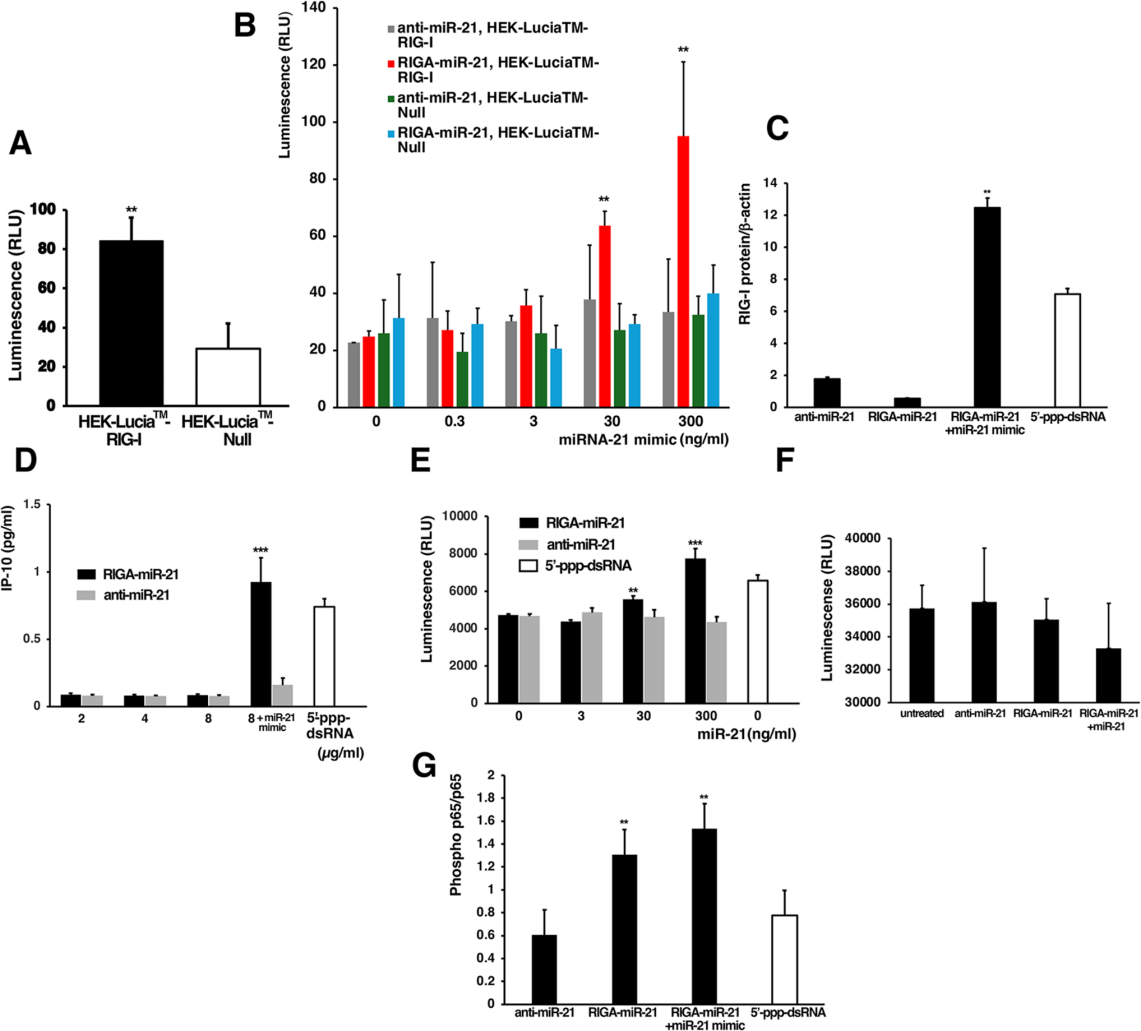
Assessment of RIG-I activation by RIGA-miR-21 in reporter cells. **A**. Validation of RIG-I mechanism using a traditional agonist 5’ ppp-dsRNA. There was a significant increase in luciferase activity in the HEK-Lucia™ RIG-I cells compared to the HEK-Lucia™ Null cells (** *p* < 0.01). **B**. Validation of the template-dependence of RIG-I activation induced by RIGA-miR-21: there was a significant RIG-I activation in HEK-Lucia™ RIG-I cells probed with RIGA-miR-21, but not with anti-miR21 ASO (** *p* < 0.01). No significant RIG-I activation was detected in HEK-Lucia™ Null cells with RIGA-miR-21 or anti-miR-21 ASO. **C**. RIG-I protein level was significantly upregulated when the cells were treated with RIGA-miR-21 in the presence of miR-21 mimic. There was no notable activation with anti-miR-21 considering the need for 5’-ppp modification for RIG-I activity. 5’ppp-dsRNA was used as a positive control. **D**. Confirmation of RIG-I activation by RIGA-miR-21 in B16-F10 melanoma cells. IP-10/CXCL-10 secretion was significantly induced by RIG-I activation in B16-F10 cells in the presence of miR-21 mimic compared to anti-miR-21 ASO with or without mimic (*** *p* < 0.001). 5’ppp-dsRNA was used as a positive control. **E**. Caspase- 3/7 activation was mediated by RIGA-miR-21 in B16-F10 cells in the presence of miR-21 template (*** *p* < 0.001). Transfection with anti-miR-21 with or without mimic did not lead to caspase-3/7 activation. 5’ppp-dsRNA was used as a positive control. **F**. There was a trend toward decrease in cell viability by RIGA-miR-21in the presence of miR-21. Treatment with anti-miR-21 did not result in viability change. **G**. Phosphorylation of the NF-κβ p65 subunit was increased in B16-F10 cell treated with RIGA-miR-21 alone or in combination with miR-21 mimic (**p < 0.01)

In our proof-of-principle studies we used unformulated RIGA-miR-21 to test whether it can activate RIG-I in miR-21 expressing human RIG-I luciferase reporter cell line, HEK-Lucia™ RIG-I, relative to the control cell line, HEK-Lucia™ Null, devoid of RIG-I. We first validated RIG-I activation in the cell lines with a traditional RIG-I agonist, 5’-ppp-dsRNA. We observed a significant increase in luciferase activity in the HEK-Lucia™ RIG-I cells compared to the HEK-Lucia™ Null cells (Fig. 2A). However, when RIGA-miR-21 was used, we could not detect RIG-I activity in these cells *in vitro* in spite of the fact that both HEK-Lucia™ RIG-I and HEK-Lucia™ Null cells express similar appreciable level of miR-21 (Supplementary Fig. S2). For that reason and to show proof-of-principle of our approach we pre-treated cells with miR-21 mimic. Figure 2B shows significant RIG-I activation in HEK-Lucia™ RIG-I cells probed with RIGA-miR-21 in a concentration-dependent manner, but not with anti-miR21 ASO. This is not surprising considering the need for 5’-ppp modification for RIG-I activity. Furthermore, no significant RIG-I activation was detected in HEK-Lucia™ Null cells with RIGA-miR-21 or anti-miR-21 ASO. Given the strict requirement for an RNA duplex for RIG-I activation [33–35] these results provided support for a template-directed mechanism of RIG-I agonism. As we show further in our *in vivo* studies, formulated RIGA-miR-21, TTX-RIGA-miR-21, was capable of activating RIG-I in tumors without the need for augmenting miR-21 expression with exogenous mimic.

Having shown in the proof-of-principle studies that RIGA-miR-21 is functional in a model cell line, we expanded our studies to the B16-F10 melanoma cell line, which is poorly immunogenic, expresses relatively low levels of miR-21 and has been used to study RIG-I signaling [36]. Untreated B16-F10 cells had relatively low levels of RIG-I, but its level was significantly increased when the cells were treated with RIGA-miR-21 in the presence of miR-21 mimic (Fig. 2C). This finding suggests that the RIG-I agonist can be effectively assembled in melanoma cells above the threshold levels of miR-21, triggering RIG-I activation and its subsequent upregulation. As expected, exposure to 5’ppp-dsRNA also significantly increased RIG-I, indicating positive feedback expression upon activation, consistent with RIG-I being a type I IFN-dependent gene. To demonstrate the induction of downstream markers of RIG-I activation, we incubated B16-F10 cells with RIGA-miR-21 with or without exogenous miR-21 mimic. The expression of IP-10/CXCL-10 protein, a member of the CXC chemokine family inducible by IFNs and linked to recruitment of activated T cells was increased after incubation with RIGA-miR-21 in the presence of miR-21 mimic but not with anti-miR-21 with or without mimic. Traditional 5’ppp-dsRNA agonist was used as a positive control (Fig. 2D). Similarly, murine triple negative breast cancer cells 4T1 expressing high levels of miR-21 compared to B16-F10 (Supplementary Fig. S4A) demonstrated similar increase in IP-10 protein compared to the cells treated with anti-miR-21 (Supplementary Fig. S4B). These results further demonstrated the 5’-ppp requirement and template dependence of RIG-I activation.

We next examined whether activation of RIG-I in B16-F10 cells led to the activation of caspase-3/7, a downstream target and a known marker of apoptosis. The results of this study showed induction of caspase-3/7 after incubation with RIGA-miR-21 in the presence of miR-21 mimic but not by anti-miR-21 with or without mimic. The assay was validated using 5’ppp-dsRNA as positive control, which substantially induced caspase-3/7 activation (Fig. 2E). Although not significant, we also observed a trend towards reduction of tumor cell viability when using RIGA-miR-21 in the presence of miR-21 compared with 5’-ppp-deficient anti-miR-21 (Fig. 2F). This effect was more pronounced and significant in 4T1 breast cancer cells with significantly higher endogenous miR-21 expression (Supplementary Fig. S4C). Collectively, these data demonstrate that RIGA-miR-21 can induce RIG-I activation through the miR-21 template in tumor cells.

Immune activation through RIG-I agonism engages the NF-κβ signaling pathway [37] with IFN regulator factor 3 (IRF-3) and phosphorylated NF-κβ p65 subunit translocating to the nucleus to induce type I IFN expression. We examined p65 protein phosphorylation in B16-F10 cells to further investigate the miR-21 dependent activation of RIG-I signaling. B16-F10 cells treated with RIGA-miR-21 showed robust p65 phosphorylation, while combination with the miR-21 mimic increased it even further (Fig. 2G). In this experiment it appeared that RIGA-miR-21 alone also elevated the phosphorylation of p65. Increased p65 phosphorylation did not coincide with elevated p65 expression (Supplementary Fig. S3), confirming the specificity of phosphorylation. These findings support the template-dependent immune response of RIG-I activation by RIGA- miR-21.

### Nanoparticle Formulation of RIGA-miR-21, TTX-RIGA-miR-21, Agonizes RIG-I in the Reporter Cell Line and Induces Apoptosis in Melanoma Cells

For translational considerations, we formulated RIGA-miR-21 with a nanoparticle carrier (TTX) using a published method [18] which involves attaching oligos to dextran-coated iron oxide nanoparticles through a disulfide bond linkage. This design allows the nanoparticles to transport therapeutic payloads into tumor cells and release upon the disulfide bond cleavage. The TTX-RIGA-miR-21 used in our study measured 21 nm in size with a polydispersity index (PDI) of 0.1 and carried 1.3 oligos per nanoparticle.

**Fig. 3:**
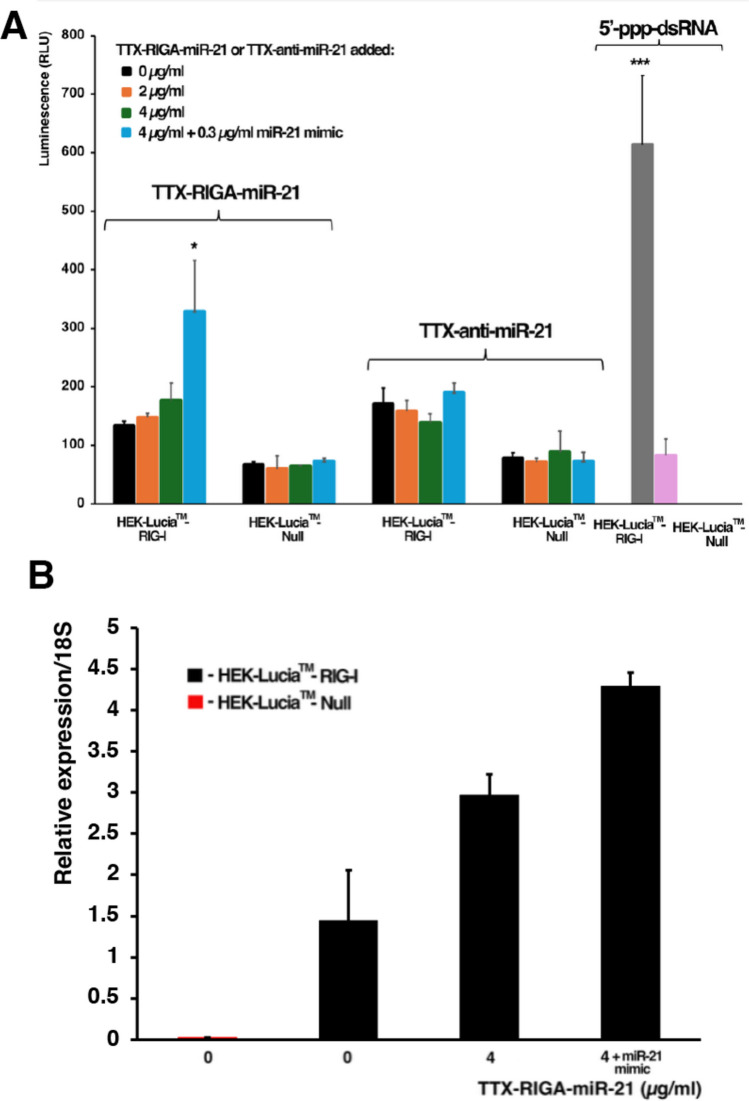
Evaluation of TTX-RIGA-miR-21-mediated RIG-I activation. **A**. Significant RIG-I activation was mediated by TTX-RIGA-miR-21 in the presence of miR-21 mimic (* *p* < 0.05). No activation was noted with TTX-anti-miR-21 with HEK-Lucia™-RIG-I or HEK-Lucia™-Null cells. 5’ppp-dsRNA served as a positive control. **B**. Relative expression of RIG-I mRNA was increased after TTX-RIGA-miR-21 treatment (* *p* < 0.05)

We first tested whether TTX-RIGA-miR-21 causes analogous activation as observed for RIGA-miRNA in the HEK-Lucia™ RIG-I reporter cell line. The results demonstrated that TTX-RIGA-miR-21 in the presence of miR-21 mimic triggered significant RIG-I activation in HEK-Lucia™ RIG-I cells, but not in HEK-Lucia™ Null cells (Fig. 3A) The 5’-ppp-deficient TTX-anti-miR-21, either alone or coupled with miR-21 mimic, did not activate RIG-I in either cell line. Traditional 5″-ppp-dsRNA agonist used as a positive control induced RIG-I activation in HEK-Lucia™ RIG-I cells, but not in HEK-Lucia™ Null cells.

Next, we assessed the impact of TTX-RIGA-miR-21 on RIG-I mRNA expression in RIG-I reporter cells by RT-qPCR analysis (Fig. 3B). In line with the results observed earlier (Fig. 2B), we verified the presence of elevated baseline levels of RIG-I mRNA in HEK-Lucia™ RIG-I cells compared to HEK-Lucia™ Null cells. Treatment with TTX-RIGA-miR-21 resulted in a notable elevation of RIG-I mRNA levels, which reached statistical significance when miR-21 mimic was added (*p* < 0.05).

**Fig. 4:**
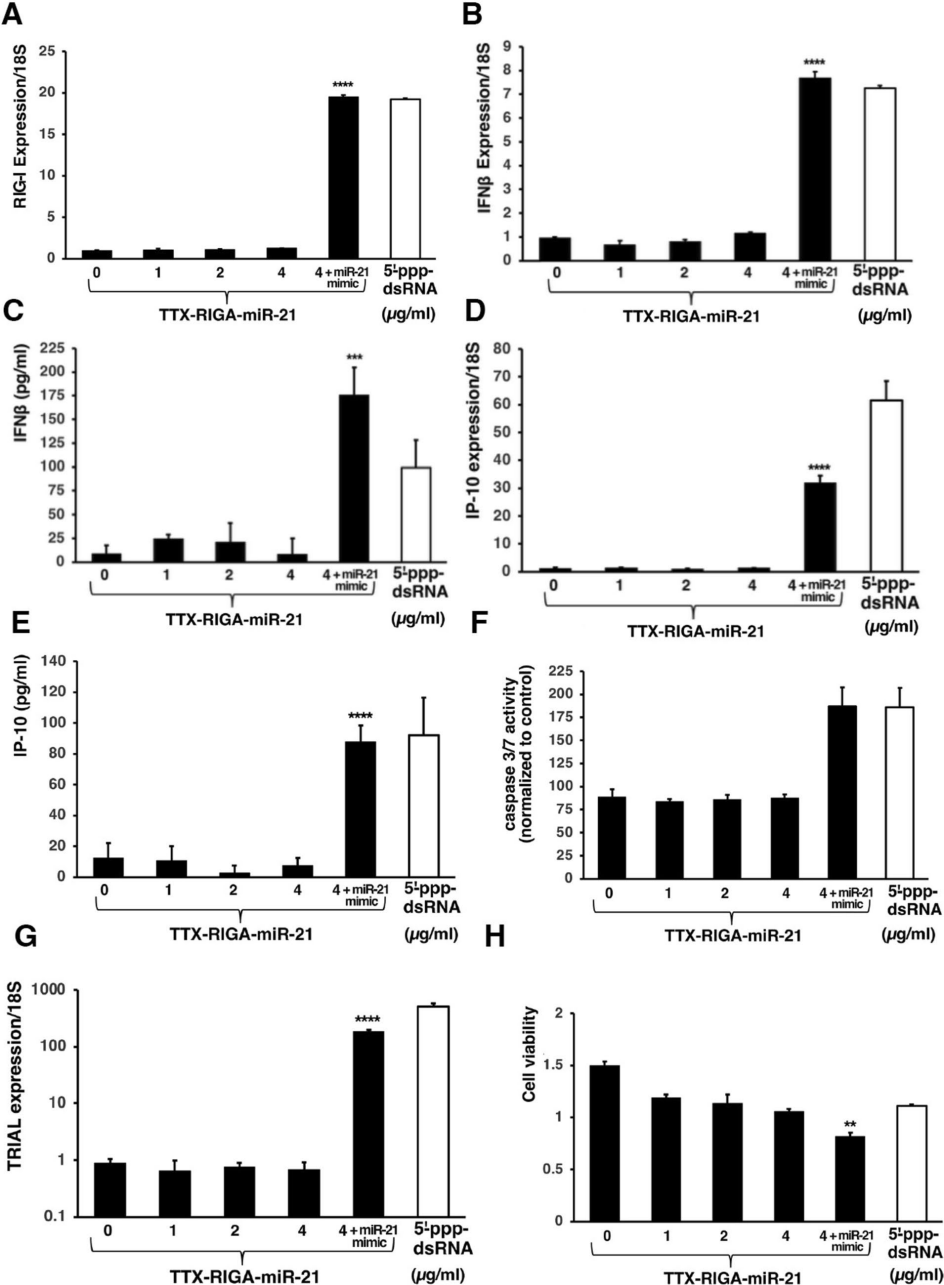
Validation of TTX-RIGA-miR-21-mediated RIG-I activation in B16-F10 melanoma cells. **A**. There was a significant RIG-I mRNA upregulation in cells treated with TTX-RIGA-miR-21 in the presence of miR-21 mimic as established by RT-qPCR analysis (**** *p* < 0.0001). **B**. A significant upregulation of IFN-β mRNA was observed in B16-F10 melanoma cells treated with TTX-RIGA-miR-21 in the presence with miR-21 mimic (**** *p* < 0.0001). **C**. A significant upregulation of IFN-β at the protein level was observed in B16-F10 melanoma cells treated with TTX-RIGA-miR-21 in the presence with miR-21 mimic (*** *p* < 0.001). **D**. A significant upregulation of IP-10 mRNA was observed in B16-F10 melanoma cells treated with TTX-RIGA-miR-21 in the presence with miR-21 mimic (**** *p* < 0.0001). **E**. We observed a significant upregulation of IP-10 at the protein level in B16-F10 melanoma cells treated with. TTX-RIGA-miR-21 in the presence with miR-21 mimic (**** *p* < 0.0001). **F**. TTX-RIGA-miR-21 caused significant caspase 3/7 activation in the presence of exogenous miR-21 (**** *p* < 0.0001). **G**. TTX-RIGA-miR-21 caused significant upregulation of TRAIL expression in the presence of exogenous miR-21 (**** *p* < 0.0001). Note: Y axe is presented as a log scale. H. TTX-RIGA-miR-21 caused significant decrease in B16-F10 cell viability in the presence of miR-21 mimic compared to untreated controls (**p < 0.01)

To validate these finding in B16-F10 melanoma cells, we next demonstrated that TTX-RIGA-miR-21 retained its ability to activate RIG-I and trigger downstream signaling effects. To this end we first investigated whether TTX-RIGA-miR-21 was able to upregulate RIG-I during activation, similar to the unformulated agonist. Indeed, we observed robust RIG-I mRNA upregulation in cells treated with TTX-RIGA-miR-21 in the presence of miR-21 mimic (Fig. 4A). Next, we confirmed significant upregulation of IFN-β in B16-F10 melanoma cells treated with TTX-RIGA-miR-21 in the presence with miR-21 mimic at both the mRNA and protein levels (Fig. 4B and C respectively). We then studied IP-10 expression at both mRNA and protein levels (Fig. 4D and E respectively). Similar to the observations with IFN-β we observed a significant increase in IP-10/CXCL10 expression at both mRNA and protein levels when the cells were treated with TTX-RIGA-miR-21 along with miR-21 mimic. Traditional 5’ppp-dsRNA served as positive control in all cases.

We next investigated whether TTX-RIGA-miR-21 could induce apoptosis in melanoma cells similar to its unformulated counterpart (Fig. 2F). To assess apoptosis induction via tumor cell-intrinsic RIG-I signaling by TTX-RIGA-miR-21, we examined caspase-3/7 activation (Fig. 4F). As before, TTX-RIGA-miR-21 caused significant activation in the presence of exogenous miR-21. As expected, traditional 5’ppp-dsRNA agonist that served as positive control also triggered robust caspase-3/7 activation. We then explored the activation of the extrinsic apoptotic pathway by measuring the expression of TNF-related apoptosis-inducing ligand (TRAIL) (Fig. 4G). Similar to our findings with caspase-3/7, we observed significantly induced TRAIL expression in cells treated with TTX-RIGA-miR-21 in the presence of miR-21 mimic. In addition, we observed significant decrease in viability in the cells treated with TTX-RIGA-miR-21 in the presence of miR-21 mimic compared to untreated controls (Fig. 4H). As expected, traditional 5’ppp-dsRNA agonist that served as positive control also triggered robust caspase-3/7 activation increase in TRAIL expression and decrease in B16-F10 cell viability.

### Therapeutic Studies With TTX-RIGA-miR-21 in B16-F10 Melanoma Tumor Model

**Fig. 5:**
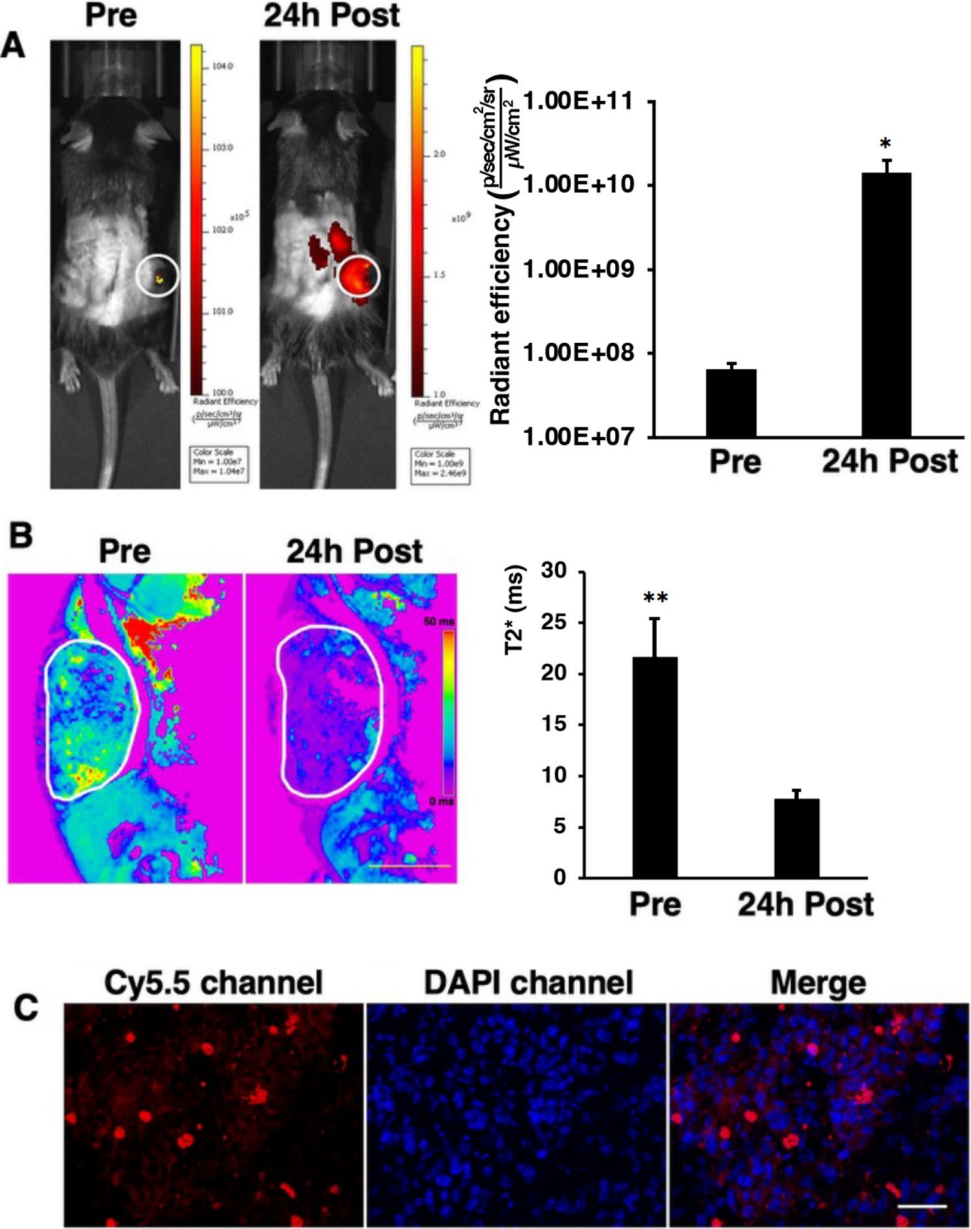
*In vivo* imaging of TTX delivery to B16-F10 melanoma model. **A**. Left: *In vivo* fluorescence optical imaging before and 24 h after intravenous injection of TTX. White circles – tumor. Right – Quantitation of the image on the left demonstrating significant increase in radiancy upon accumulation of the nanoparticles (p = 0.3). Different scaling in pre- and post-images reflect several orders of magnitude increase in signal in post-images. **B**. Left: *In vivo* MRI before (at baseline) and 24 h after intravenous injection. Tumor is outlined in white. Right: Quantitation of the image on the left demonstrating significant drip in T2* i upon accumulation of the nanoparticles (p = 0.01). Scale bar = 5 mm. **C**. Ex vivo correlative fluorescence microscopy confirming the presence of Cy5.5-labeled nanoparticles in the tumor tissue. Red – Cy5.5, blue – DAPI nuclear stain. Scale bar = 50 µm

Delivery of Cy5.5-labeled TTX was assessed using *in vivo* optical and magnetic resonance imaging before and 24 h after intravenous injection in B16-F10 tumor-bearing mice. Figure 5A demonstrates the accumulation of Cy5.5-labeled nanoparticles in the tumor region, indicated by the increase in fluorescence signal from the baseline (6.41 × 10^7^ ± 1.2 × 10^7^ vs 1.41 × 10^10^ ± 6.27 × 10^9^; p = 0.3). MRI of T2* maps (Fig. 5B) demonstrated significant drop in T2* value 24 h after injection (from 21.58 ± 3.94 to 7.73 ± 0.98; p = 0.01), which is characteristic for superparamagnetic nanoparticles and in line with our previous results [17–19]. Pre- and post- contrast T2*-weighted images are shown in Supplementary Fig. S5. Ex vivo fluorescence microscopy showed the presence of nanoparticles in the tumors confirming the delivery (Fig. 5C).

**Fig. 6:**
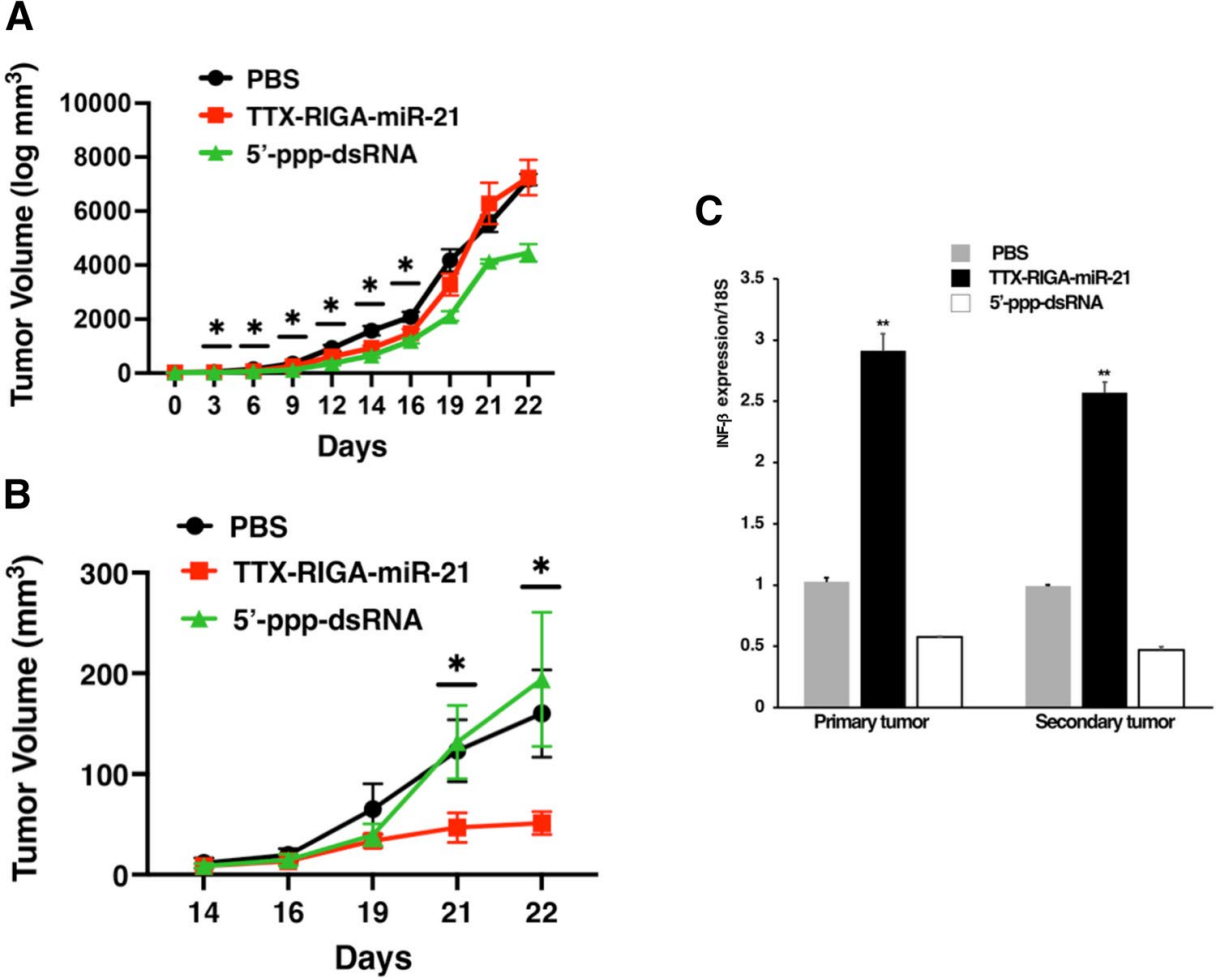
TTX-RIGA-miR-21 inhibits tumor growth and induces strong anti-tumor immunity in B16-F10 allografts. Study design is shown in Supplementary Fig. S1. **A**. A significant reduction in primary tumor volume was observed, lasting up to 5 days following cessation of treatment (*p < 0.05). A close-up figure of Days 0–16 is shown in Supplementary Fig. S6. **B**. Secondary tumor growth was significantly inhibited by TTX-RIGA-miR-21 compared to the PBS and 5’-ppp-dsRNA groups (* *p* < 0.05). **C**. A significant upregulation of IFN-β was observed in the group treated with TTX-RIGA-miR-21 in both primary and secondary tumors compared to the groups treated with PBS or 5’-ppp-dsRNA (* *p* < 0.05)

In our therapeutic studies (study design in shown in Supplementary Fig. S1) injections of TTX-RIGA-miR-21 (i.v.), PBS (i.v.) or 5’-ppp-dsRNA (intratumorally) started on Day 4 after tumor implantation and continued for 7 days until Day 11 and then were discontinued. Figure 6A demonstrated that TTX-RIGA-miR-21 treatment led to a significant reduction in primary tumor growth relative to the PBS control group. This effect persisted until Day 16 (5 days after treatment cessation), after which tumor progression gradually resumed. A close-up figure depicting Days 0–16 is shown in Supplementary Fig. S6. As expected, intratumorally delivered 5’-ppp-dsRNA suppressed primary tumor growth throughout the study. In contrast to the primary tumor, the growth of secondary tumor injected on Day 15 was remarkably inhibited in the TTX-RIGA-miR-21 group while it remained rapid in both the PBS and 5’-ppp-dsRNA groups (Fig. 6B). We also found a significant IFN-β upregulation in the TTX-RIGA-miR-21 group in both primary and secondary tumors (Fig. 6C). These in vivo observations contrasted sharply with the *in vitro* results obtained with TTX-RIGA-miR-21 that heavily relied on the presence of miR-21 mimic (Figs. 4B, C). In addition, 5’-ppp-dsRNA did not upregulate IFN-β not only in the secondary tumor but also in the primary tumor with ongoing antitumor activity, despite demonstrating this effect *in vitro* (Figs. 4B, C). The likely immunological mechanisms behind the rechallenge study include immune memory formation likely involving CD8 + T cells and type I INF-driven priming (Fig. 6C) within the tumor microenvironment. We plan to investigate these effects further in various cancer models.

## Discussion

While immunotherapies have revolutionized cancer treatment, their clinical applications are hampered by limited effectiveness and/or prohibitive costs. Activation of RIG-I in cancer cells can ‘heat up’ the TME by activating immune cells leading to induction of immunogenic cell death (ICD) and releasing tumor antigens within the TME and thus converting cancer cells into potential cellular vaccines. These characteristics make RIG-I an exceptionally appealing candidate for cancer treatment for eliciting broad and persistent antitumor immunity. However, challenges such as off-target effects and delivery issues have hindered the full potential of this approach. The issue of off-target effects exists because of the ubiquitous expression of RIG-I-like receptors in the body [6, 7]. Since the delivery of double-stranded agonists is non-specific, any cell that receives the agonist can trigger the response causing significant toxicity and unintended systemic immune activation. The issue of delivery was somewhat mitigated in recent clinical trials [10] and preclinical studies [6–9] by the use of the cationic polymeric transfection reagent jetPEI®, which electrostatically condenses nucleic acids [38]. Other delivery methods in development include PLGA polymers [12], lipid calcium phosphate [13], lipid-based nanoparticles [14], and extracellular vesicles [15]. However, in addition to the challenges associated with these delivery methods such as toxicity, lung accumulation and cytosolic release [39] all these studies used double-stranded constructs, resulting in non-specific activation of RIG-I.

To resolve the issue of non-specific RIG-I activation we proposed to utilize a short single-stranded RNA oligonucleotide with 5’-triphosphate modification, a RIG-I agonist precursor that upon entry to cells hybridizes with an endogenous single-stranded miRNA to form an active double stranded RIG-I agonist. MiRNAs represent particularly attractive targets due to their similar length to 5’-ppp-dsRNA agonists and frequent upregulation in tumors. The most advantageous aspect of this approach is its highly specific activation of RIG-I, occurring only in cells where double-stranded RNA is formed, thereby avoiding off-target effects associated with the delivery of exogenous double-stranded constructs. RIG‑I activation in the proposed model was supported by phosphorylation of NF‑κB p65 (Fig. 2G) and induction of ISG-associated cytokines, consistent with canonical RIG‑I signaling.

The second issue – effective delivery of oligonucleotides to the cells of interest—has not been resolved for the RIG-I approach either. In general, delivering an active oligonucleotide molecule to tumors represents a significant challenge due to its inability to cross the cell membrane because of charge-charge repulsion [40], its short half-life and digestion by nucleases [41, 42], entrapment in endosomes [43], off-target effects, and activation of unwanted immune responses [44]. In the study presented here we overcame this issue by utilizing the TTX nanoparticle platform that has previously been used by us for delivery of antisense oligos and siRNAs in small and large animals in several cancer types to silence specific biomarkers [17–23]. The iron oxide-based nanoparticles (TTX platform) used in this study are small (21 nm) and long-circulating and distribute to tumors via the well-established enhanced permeability and retention (EPR) effect [18]. Once in the interstitium, they are taken up by the tumor cells via micropinocytosis based on the high metabolic activity of the tumor cells [45]. While we have previously used these nanoparticles for oligonucleotide delivery and target silencing in various cancer models in small and large animals [17–19, 23], this is the first time when we applied this delivery platform for cancer immunotherapy. Significant advantages of this platform include its well-characterized pharmacokinetics, absence of systemic toxicity, and proven biocompatibility. Importantly, this platform confers the ability to deliver a payload using image guidance adding to its translational value. Due to magnetic properties of the nanoparticle core and versatility afforded by the aminated dextran coat allowing for conjugation of secondary imaging reporters (such as Cu64 and Cy5.5) it can be used with clinically relevant modalities (MRI and PET) as well as with laboratory development imaging tools (near infrared optical imaging). Imaging can be employed clinically to monitor delivery during therapeutic development and subsequently during patient treatment. Indeed, to confirm nanoparticle delivery in the present study, we employed magnetic resonance imaging (MRI), supported by correlative optical imaging and validated through fluorescence microscopy.

In these studies, we synthesized RIG-I agonist (TTX-RIGA-miR-21) targeting miR-21 commonly overexpressed in tumor cells [24, 46, 47]. We demonstrated its ability to activate RIG-I, upregulate the expression of inflammatory cytokines, induce apoptosis and decrease in viability in melanoma cells *in vitro* and *in vivo*. Importantly, in our *in vivo* studies we demonstrated an abscopal effect characteristic for classical RIG-I agonists injected intratumorally [8]. While the amount of TTX-RIGA-miR-21 delivered via systemic injection was probably substantially lower than the intratumorally delivered 5’-ppp-dsRNA [48] we still observed encouraging results presumably extending from RIG-I activation in the tumor microenvironment (TME) and affecting both cancer and non-cancer cells. Despite the intratumoral injection 5’-ppp-dsRNA did not show significant effect on the tumor growth likely due to rapid nuclease degradation, inadequate dispersion within tumor tissue, and limited cytosolic entry without a delivery vehicle. Most importantly, while in our *in vitro* studies we had to rely on exogenous miR-21, it was not needed in our animal studies where RIG-I activation was clearly detected and caused tumor growth retardation and IFN-β production. The reason for this difference could be because the formulated agonist has a relatively long half-life *in vivo* allowing for IFNs to accumulate and elicit miR-21 expression as has been shown before [49]. This assumption is supported by the increased IFN-β response in both primary and secondary tumors in animals treated with TTX-RIGA-miR-21 (Fig. 6C). In addition, in our *in vitro* studies we noticed a trend toward increased RIG-I mRNA expression and reduced cell viability when formulated RIGA-miR-21 was used (Figs. 3B and 4H). In our future studies, we will expand template-directed RIG-I activation screening across multiple miR-21–expressing cancer cell lines, including triple-negative breast cancer, pancreatic ductal adenocarcinoma, and glioblastoma. These models exhibit high endogenous miR-21 levels and represent clinically relevant tumor types where immune-cold microenvironments limit current immunotherapies.

## Conclusion

In summary, RIG-I activation holds promise for enhancing tumor immunogenicity in cancer immunotherapy and warrants further evaluation. The use of image-guided nanoformulated RIG-I agonist demonstrated its potential for future clinical translation given that related formulations are already in clinical trials. It could potentially complement existing strategies including CAR T cell and anti-PD-1/PD-L1 antibody. We believe that enabling tumor-selective innate immunity activation through this model is a significant step toward effectively using RIG-I in clinical cancer therapy.

## Supplementary Information

Below is the link to the electronic supplementary material.

ESM 1 (PDF 1.82 MB)

## Data Availability

All research data and computer codes are available from the corresponding author upon request.
