# Supplementary material for: Template-Directed RIG-I Agonist Assembly for Image-guided Targeted Cancer Immunotherapy
Source: Mol Imaging Biol. 2026 Feb 19;28(2):320–33. doi: 10.1007/s11307-026-02087-8 (PMC13160977; doi:10.1007/s11307-026-02087-8)
Supplement: Supplementary file 1 — (PDF 1.82 MB) [file 11307_2026_2087_MOESM1_ESM.pdf]

Supplementary Materials for

Template-Directed RIG-I Agonist Assembly for Image-guided Targeted Cancer Immunotherapy

Subrata K. Ghosh[1], Douglas Lazarus[2], Neil Robertson[1], Qiyong P. Liu[1], Elizabeth Kenyon[3,4], Christian L. Mallett[3,5], Ming Chen[3,4], Zdravka Medarova[1*] and Anna Moore[3,4*]

[1]TransCode Therapeutics, Inc., 400 Trade Center, Suite 5900, Woburn, MA 01801
[2]Avastus Preclinical Services, 44 Spinelli Place, Cambridge, MA 02138
[3] Precision Health Program, Michigan State University, 766 Service Rd., East Lansing, MI 48224
[4]Department of Radiology, College of Human Medicine, Michigan State University, 846 Service Rd., East Lansing, MI 48224
[5]Institute for Quantitative Health Science and Engineering, Michigan State University, 775 Woodlot Dr., East Lansing, MI 48224

* Correspondence should be addressed to Anna Moore (moorea57@msu.edu) or Z. M. (zdravka.medarova@transcodetherapeutics.com)

Anna Moore, Ph.D., Precision Health Program, Michigan State University, 766 Service Rd, East Lansing, MI 48824 USA; Tel: 617-584-2947: E-mail: moorea57@msu.edu
Zdravka Medarova, Ph.D., TransCode Therapeutics, Inc., 400 Trade Center, Suite 5900, Woburn, MA 01801, USA; Tel: 508-304-7603; E-mail: zdravka.medarova@transcodetherapeutics.com

Supplementary Materials and Methods

Cell transfection

For transfection, appropriately diluted oligos were mixed with 100 μL of LyoVec™ cationic lipid transfection agent (Catalog No, lyec-12; InvivoGen) and incubated at 15 – 25°C for 15 min to 1 h with the cells. Ten μL of the LyoVec^{TM}/oligo complex was added to 200 μL of culture media. Luciferase activity in supernatants was measured after 48 hours as indicated below.

For validation of the template-dependence of RIG-I activation induced by RIGA-miR-21, HEK-Lucia™ RIG-I and HEK-Lucia™ Null cells were transfected with RIGA-miR-21 or anti-miR-21 along with increasing concentrations of miR-21 mimic or 5'ppp-dsRNA.

Nanoparticle synthesis and characterization

Iron oxide nanoparticles were synthesized with proprietary modifications based on the protocol described in [17, 18]. Briefly, $FeCl_3.6H_2O$ and $FeCl_2.4H_2O$ were added to Dextan-T10 (Pharmacosmos, Holbaek, Denmark) while flushing argon gas into the reaction mixture followed by the addition of cold NH_4OH. The resulting dextran-coated MNs were cross-linked and aminated with subsequent addition of NaOH, concentrated epichlorohydrin and concentrated NH_4OH. The nanoparticle solution was purified using a dialysis bag against water and 20mM citrate buffer pH 8.0. For near-infrared optical imaging and correlating fluorescence microscopy, nanoparticles were labeled with NIR dye Cy5.5 mono-reactive NHS ester (GE Healthcare, Piscataway, NJ, USA) according to the standard protocol. Cy5.5-labeled magnetic nanoparticles were then conjugated to the corresponding activated oligos through the heterobifunctional linker N-succinimidyl 3-[2-pyridyldithio]-propionate (SPDP; Thermoscientific Co.). Nanoparticle size was determined by the Dynamic light scattering (DLS, Zetasizer Nano ZS; Malvern Instruments Ltd, Malvern, Worcestershire, UK) and showed a hydrodynamic diameter of about ~21 nm (DLS, PDI 0.1). Iron concentration was determined using a colorimetric assay involving nanoparticle digestion with HCl, reduction with ascorbic acid, neutralization with NaOAc, color development with o-phenanthroline, and quantification at 510 nm. To determine the oligo content in the nanoparticle formulations, the particles were treated with TCEP and NH_4HCO_3 to release the oligos. The liberated oligos were separated by ultrafiltration and quantified at 260 nm.

Western Blot

To assess protein expression of IFN-β, IP-10 and RIG-I we performed Western blot with corresponding antibodies. Cells were washed and lysed on the plate in Pierce™ IP Lysis Buffer (Catalog No. 87787; ThermoFisher Scientific) with Halt Protease and phosphatase inhibitors (Catalog No. 78440, ThermoFisher Scientific) on ice for 15 min. Lysates were centrifuged (14,000 x g, 4°C, 10 min). Proteins were quantified using the Quick Start Bradford Protein assay kit (Catalog No. 5000202; Bio-Rad, Hercules, CA), electrophoresed (50 μg) on a 4-20% Mini-Protean TGX Stain-Free Protein Gel (Bio-Rad Laboratories, Hercules, CA), and transferred to nitrocellulose membranes. The membranes were blocked for 1 h at room temperature (RT) with Blocker Blotto in TBS (Catalog No. 37530, ThermoFisher Scientific), incubated overnight at 4°C with rabbit monoclonal antibody (1:1000 dilution, Cell Signaling Technology, MA) to RIG-I (Catalog No. 3743), 2hosphor-p65 (3033), p65 (8242), or reference protein β-actin (5125), followed by incubation with anti-rabbit IgG (secondary antiserum), horseradish peroxidase-conjugated secondary antibodies (1:2000 dilution; 7074) for 2 h at 37°C. Proteins were detected using Pierce ECL Plus Western Blotting Substrate (Catalog No. 32132; ThermoFisher Scientific)

and the Invitrogen IBRIGHT CL750 imaging system. Quantification of protein bands in the Western blots was performed using NIH Image J software.

RT-qPCR

Total miRNA was purified from cells using miRNeasy Mini Kit (Catalog No. 217004; Qiagen, Germantown, MD). Complementary DNA (cDNA) was synthesized using miRCURY LNA RT Kit (Catalog No. 339340, Qiagen). MiR-21 was quantified using the miRCURY LNA SYBR Green PCR Kit (Catalog No. 339345, Qiagen) and primers from miRCURY LNA miRNA PCR Assays (Catalog No. 339306, Qiagen) for hsa-miR-21-5p (GeneGlobe ID YP00204230). Reactions were run on StepOnePlus Real-Time PCR System (Applied Biosystems; ThermoFisher Scientific) using the following cycling program: 2 min at 95°C and 2-step cycling (40 cycles) of denaturation (10 s at 95°C), and combined annealing/extension (60 s at 56°C). The calculation of relative expression was performed using the $2^{-\Delta\Delta Ct}$ method. U6 small nuclear RNA (GeneGlobe ID YP02119464) was used as a reference.

To quantify mRNA, total RNA was extracted from cells using the PureLink RNA Mini Kit (ThermoFisher Scientific). Single-stranded cDNA was synthesized using a High-Capacity cDNA Reverse Transcription Kit (ThermoFisher Scientific), which was analyzed by quantitative PCR using PowerUp™ SYBR™ Green Master Mix (ThermoFisher Scientific). Reactions were run on StepOnePlus Real-Time PCR System following conditions described above for miRNA and the relative expression was calculated similarly. The primers used are shown in Supplementary Table 1.

Supplementary Table 1. Primers and probes used in the study.

Gene	Oligonucleotide	Sequence
RIG-I (human)	Forward primer	5'-GGACGTGGCAAAACAAATCAG-3'
	Reverse primer	5'-GCAATGTCAATGCCTTCATCA-3'
GAPDH (human)	Forward primer	5'-ATGACATCAAGAAGGTGGTG-3'
	Reverse primer	5'-CATACCAGGAAATGAGCTTG-3'
RIG-I (mouse)	Forward primer	5'-GAGAGTCACGGGACCCACT-3'
	Reverse primer	5'-CGGTCTTAGCATCTCCAACG-3'
TRAIL (mouse)	Forward primer	5'-ATGGTGATTTGCATAGTGCTCC-3'
	Reverse primer	5'-GCAAGCAGGGTCTGTTCAAGA-3'
IFN-β (mouse)	Forward primer	5'-AGGGCGGACTTCAAGATC-3'
	Reverse primer	5'- CTCATTCCACCCAGTGCT-3'
IP-10/CXCL-10 (mouse)	Forward primer	5'-AATGAGGGCCATAGGGAAGC-3'
	Reverse primer	5'-AGCCATCCACTGGGTAAAGG-3'
18S (mouse)	Forward primer	5'-GTTCCGACCATAAACGATGCC-3'
	Reverse primer	5'-TGGTGGTGCCCTTCCGTCAAT-3'

In vivo imaging of tumoral delivery

Mice (C57BL/6J, 8-week-old) were obtained from the Jackson Laboratory. B16-F10 melanoma cells (1 x 10^5) were subcutaneously injected into the right flank. To assess the delivery, mice were imaged using in vivo fluorescence and magnetic resonance imaging (FLI and MRI). FLI tumor-

bearing mice were scanned in Cy5.5 channel before and 24 hrs after intravenous injection of the nanoparticles using an In Vivo Imaging System (IVIS Spectrum, Revvity, Waltham, MA). For the analysis of therapeutic accumulation in cancer lesions, ROIs were drawn around each tumor using LivingImage 4.5 software. MRI was performed following FLI sessions on the same day, using a 7T MR scanner (BioSpec 70/30, Bruker, Billerica, MA), the mice were on their right sides on top of a 4x4 cm 4-channel array surface coil with the tumor in the center. An 86 mm volume coil was used as the transmit coil. Mice were kept warm by a circulating warm water pad and breathing and temperature were monitored. The following parameters were used for T2star sequences: 3D T1FLASH: TR/TE 30/8, 5 averages, field of view 20x10x15 mm, matrix size 200x100x120, resolution 100x100x125 um, flip angle 12 degrees, 33-minute scan time. In addition, T2* maps were collected with the following parameters: a multi gradient echo image with TR/TE: 800/3.5 ms, 10 echo images with 5 ms echo spacing and positive readout echoes, 2 averages, 5 800 um axial slices, field of view 20x20cm, matrix 128x128, resolution 156x156 um, scan time 2.5 minutes. All images were acquired in the center of the tumor. Maps were analyzed using the image sequence analysis module in Paravision 360 v3.5.

Supplementary Figures

Supplementary Figure S1.

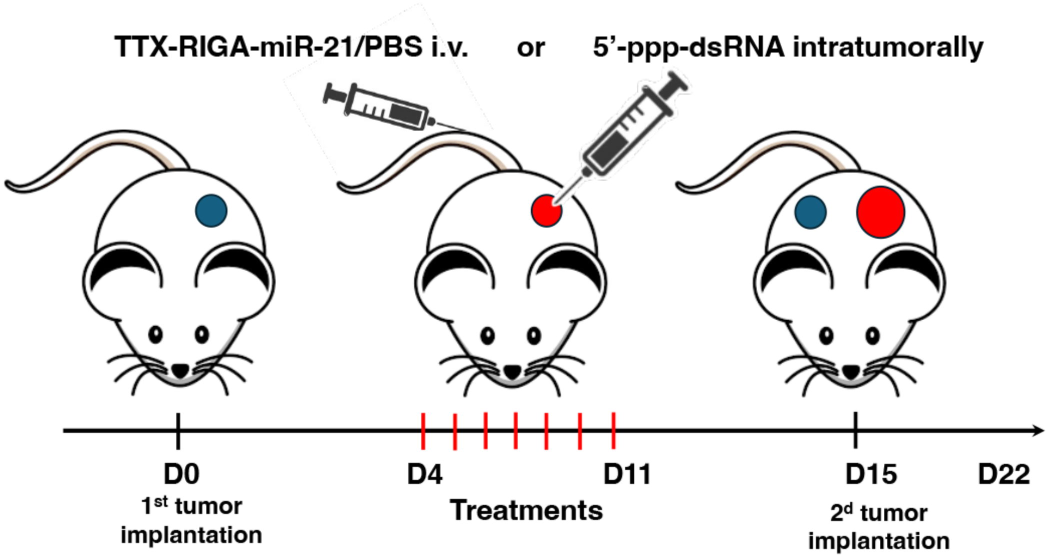

Study design in the animal model. Primary tumors were generated by subcutaneous injections with B16-F10 melanoma cells on Day 0 (D0). Daily treatments with TTX-RIGA-miR-21 (i.v.), PBS (i.v.) or 5'-ppp-dsRNA (intratumorally) started on Day 4 after tumor implantation and continued for 7 days. On Day 15, secondary tumors on the opposite side were generated by subcutaneous injection of B16-F10 cells. The tumor development was monitored for an additional 7 days (until D22) without resuming treatment.

Supplementary Figure S2

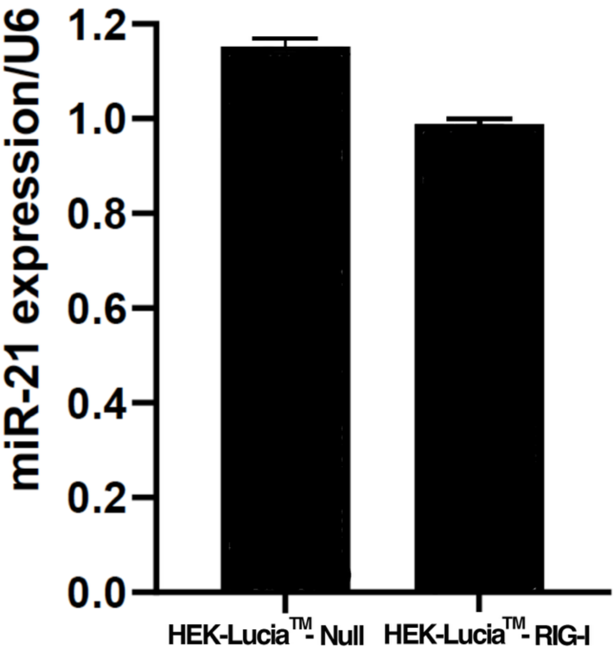

HEK-Lucia™ RIG-I and HEK-Lucia™ Null cells express similar appreciable level of miR-21.

Supplementary Figure S3.

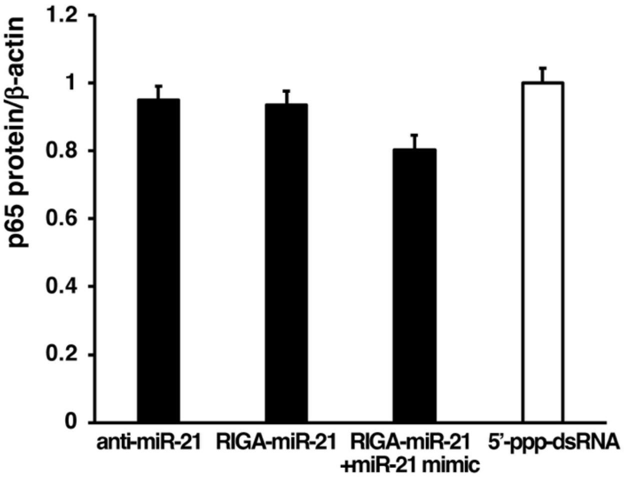

RIGA-miR-21-induced phosphorylation did not coincide with elevated p65 expression, confirming the specificity of phosphorylation.

Supplementary Figure S4.

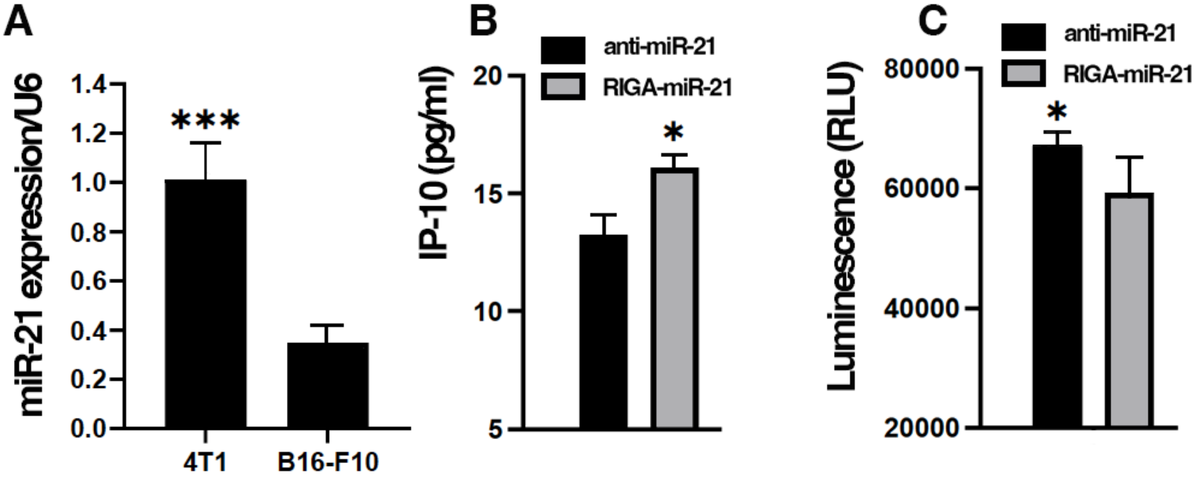

A: Endogenous miR-21 expression in 4T1 and B16-F10 cells. The data presented are ΔΔ*CT* as mean ± SD (*** $p < 0.001$). B: IP-10 induction mediated by RIG- I activation in 4T1 cells. C: Cell viability was reduced by RIGA-miR21 in 4T1 cells. B and C effects were RIG-I agonist specific, since they were not seen with the ppp-deficient anti-miR-21.

Supplementary Fig. S5.

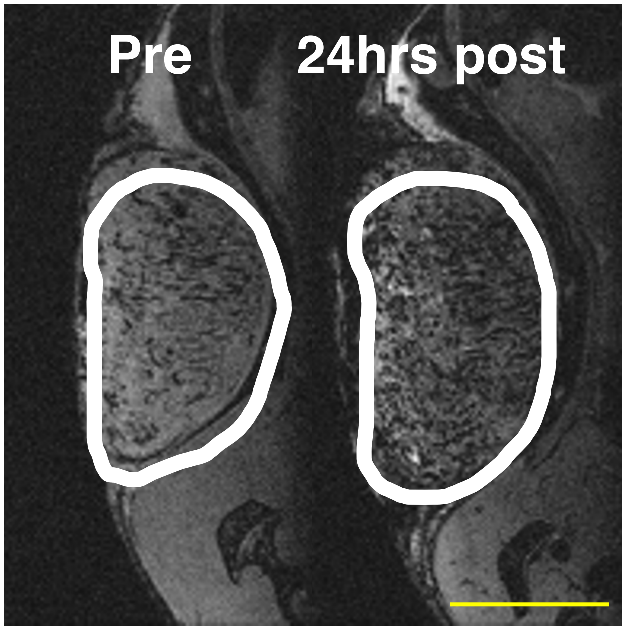

T2*-weighted images of B16F10 melanoma tumor demonstrating decrease in signal intensity 24 hrs post intravenous injection of the agent. Tumor is outlined in white. Scale bar = 5 mm.

Supplementary Fig. S6.

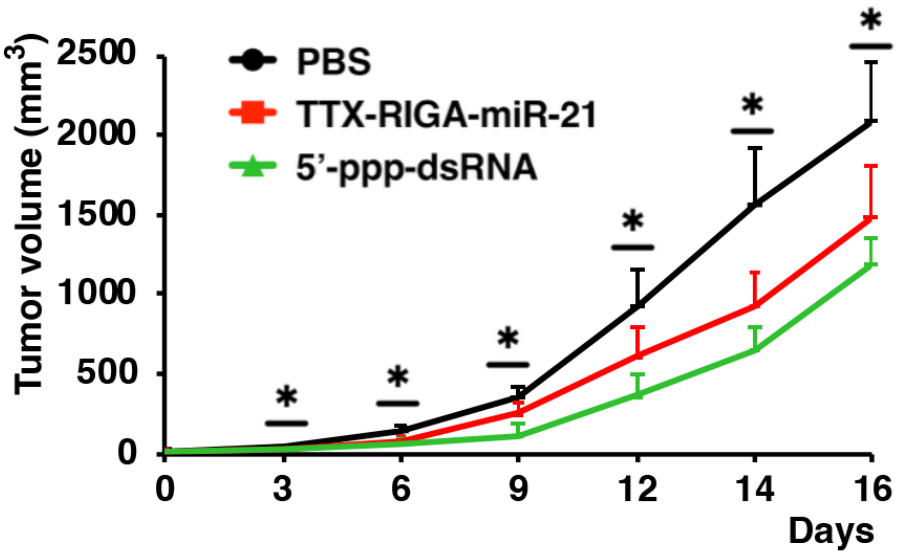

Supplementary to Fig. 6, this figure demonstrates that TTX-RIGA-miR-21 inhibits tumor growth and induces strong anti-tumor immunity in B16-F10 allografts. A significant reduction in primary tumor volume was observed, lasting up to 5 days following cessation of treatment (*$p < 0.05$). Days 0-16 are shown.
